# Combined Detection of IFN-γ and Lymphocyte Subsets with Activation Indicators in the Clinical Application of Mycobacterium Tuberculosis Infection at Different Times

**DOI:** 10.1007/s00284-023-03306-z

**Published:** 2023-04-27

**Authors:** Yiwen Chen, Lahong Zhang, Changjun Zhou, Yuhua Liu, Feng Pan, Qiang Ke, Zhaojun Chen

**Affiliations:** 1 Clinical Laboratory Department, Hangzhou Normal University Affiliated Hospital, Zhejiang Province, Hangzhou, 310015 China; 2 Hangzhou Normal University Affiliated Hospital (Clinical College), Hangzhou, China

## Abstract

The immune status of *mycobacterium tuberculosis* (*MTB*) infection is essential for the diagnosis and treatment of this disease. In this work, we aim to evaluate the clinical significance of the combination of serum IFN-γ, IGRAs (Interferon-Gamma Release Assay), lymphocyte subset with activation indicators detection in active and latent tuberculosis infection patients. For this study, anticoagulant whole blood were collected from 45 active tuberculosis (AT group), 44 latent tuberculosis (LT group) and 32 healthy controls (HCs group). The serum IFN-γ and IGRAs detected by chemiluminescence, and the percentage of lymphocyte subsets and activated lymphocytes detected by flow cytometry. The results showed combined IGRAs, serum IFN-γ and NKT cells not only has good diagnostic efficiency for the AT, but also provides a laboratory diagnostic method to distinguish AT from LT. Activation indicator of CD3^+^HLA-DR^+^T and CD4^+^HLA-DR^+^T can effectively distinguish LT from HCs. While combined CD3^+^T, CD4^+^T, CD8^+^CD28^+^T, Treg and CD16^+^CD56^+^CD69^+^ cells can distinguish AT from HCs. This study showed combined direct detection of serum IFN-γ and IGRAs as well as lymphocyte subsets with activation indicators which may provide laboratory basis for the diagnosis and differential diagnosis of active and latent *MTB* infection.

## Introduction

*Mycobacterium Tuberculosis* (*MTB*) is one of the top 10 highest mortality rates worldwide [1]. In the early stage of tuberculosis infection, most people's immune defense mechanism will protect [2], but *MTB* as an intracellular parasite can form latent tuberculosis infection (LTBI) through a complex immune escape mechanism [3]. According to WHO data, 5% to 10% of the population of LTBI can convert into active tuberculosis infection (ATBI) [4]. The increased incidence of chronic diseases in recent years has led to a decline in patient’s body immunity, and the risk of developing LTBI patients into ATBI has been further increased [5]. Therefore, the diagnosis and regular monitoring of patients with LTBI is also the key to effectively reduce the incidence of tuberculosis and control the development and transmission of tuberculosis [6, 7]. However, it is still difficult to diagnose LTBI, because the *MTB* are mostly dormant in the body.

The main protective mechanism of *MTB* infection is cellular immunity [8]. The immune response against *MTB* infection is determined by a network of lymphocytes, mononuclear macrophages and their related cytokines [9]. The difference of immune function of *MTB* infected patients can lead to different states of infection, using flow cytometry to detect the percentage of lymphocyte subsets with their activation indicators in different states of infection, through analyzing the expression of immune cells in ATBI and LTBI, can improve the sensitivity of diagnosis and differential diagnosis of active and latent *MTB* infection.

IFN-γ is mainly formed by the activated CD4^+^T, CD8^+^T and NK cells which are produced by activating monocyte macrophages, inducing macrophage maturation, clearing *MTB* infected cells, and mediating late-onset hypersensitivity [10]. IFN-γ plays a key role in the immune reaction of anti-*MTB* infection. Studies have shown that the level of serum IFN-γ is correlated with *MTB* disease activity. Under normal conditions, T cells can secrete less IFN-γ, but the peripheral blood mononuclear cells of tuberculosis patients will produce high concentrations of IFN-γ after *MTB* stimulation. After effective drug treatment, the level of IFN-γ decreases. IGRAs is designed to use *MTB*-specific activated T cells present in peripheral blood mononuclear cells of *MTB* infected patients to secrete IFN-γ after stimulation with *MTB* specific antigen. IGRAs have been used as an auxiliary means in the diagnosis of *MTB* infection, due to their convenient detection, rapid diagnosis and quantitative detection [11], this assay recommended by the WHO and the CDC [12].

This study is proposed to provide a laboratory basis for the diagnosis and differential diagnosis of ATBI and LTBI which combined direct detection of serum IFN-γ and IGRAs as well as lymphocyte subsets with activation indicators.

## Materials and Methods

### Sample Collection

A total of untreated 45 ATBI patients, 44 LTBI patients and 32 HCs patients admitted to the Affiliated Hospital of Hangzhou Normal University from October 2021 to November 2021 were selected as the study subjects. There were no significant differences in age and gender in group 3 (all *P* > 0.05) (Table 1).

**Table 1:** Demographic characteristics of patents

characteristic	AT (*N* = 45)	LT (*n* = 44)	HCs (*n* = 32)	P value
Age	44.4 ± 15.3	51.2 ± 16.7	43.7 ± 7.9	*P* > 0.05^a^
Sex				
Male	30 (66.7%)	28 (63.6%)	21 (65.6%)	*P* > 0.05^b^
Female	15 (33.3%)	16 (36.4%)	11 (34.4%)	*P* > 0.05^b^

^a^P values were calculated by Mann–Whitney test for continuous variables

^b^P values were calculated by Fisher’s exact test for categorical variables

### Diagnostic Criteria

The samples enrolled in this study from patients with MTB infection were divided into clinically ATBI patients and LTBI patients. According to the diagnostic criteria for tuberculosis [13] and tuberculosis classification criteria [14]. ATBI meet the following conditions: (1) obvious clinical symptoms such as low fever, cough, sputum, hemoptysis; (2) positive pathogens or positive histopathology to exclude other non-tuberculous lung diseases. LTBI meet the following conditions: (1) positive IGRAs experiments; (2) negative bacterial experiments and imaging of *MTB*; and (3) no obvious clinical symptoms.

HCs meet the following conditions: born BCG vaccination; chest X-ray exposure was normal; no recent exposure Tuberculosis patients no acute, chronic disease, allergy and immunity disease.

All enrolled patients were excluded from the use of hormones, immunosuppressants, or other HLA-DR drugs affecting immune function and excluding diseases such as AIDS, autoimmune disease, or tumor.

### Lymphocyte Subset and Activation Indicator Detection

Samples of EDTA anticoagulated peripheral blood (5 mL) were collected from three groups’ patients. All samples were tested within 6 h of being obtained. Briefly, CD3^+^/CD4^+^/CD8^+^ T-cell, CD19^+^ B-cell, CD3^−^CD16^+^CD56^+^ NK-cell, CD3^+^CD16^+^CD56^+^ NKT-cell, CD4^+^CD25^+^CD127^−^ Treg-cell, CD3^+^HLA-DR^+^ T-cell and CD4^+^HLA-DR^+^ T-cell, CD8^+^ CD28^+^, and CD16^+^CD56^+^CD69^+^ counts (%) were measured by multiple-color flow cytometry with human monoclonal anti-CD3-FITC, anti-CD4-PE, anti-CD8-APC, anti-CD19-PE, anti-CD16-FITC, anti-CD56-APC, anti-CD25-PE, anti-CD127-BV421, anti-HLA-DR-Alexa700, anti-CD28-BV510 and anti-CD69-PE antibodies (BD Multitest) according to the manufacturer’s instructions. The cells were analyzed by flow cytometry system (Becton Dickinson, USA) according to the operating procedure.

### Serum IFN-γ and IGRAs Detection

#### IGRAs

3 mL of the patient's elbow venous blood from three groups was extracted and injected into heparin sodium anticoagulant tube, and 1 mL of anticoagulant blood was added into N culture tube (background control), P culture tube (non-specific stimulation of original plant hemagglutinin) and T culture tube (tuberculosis specific antigen ESAT-6 and CFP-10) containing different stimulants, respectively. Then, the culture tubes were rapidly placed in a constant temperature water bath at 37℃ for 22–24 h. The supernatant was centrifuged and the content of IFN-γ in serum was tested by direct chemoluminescence immunometric assay.(XMUMIC, China).

### Serum IFN-γ Direct Detection

3 mL samples of EDTA anticoagulated peripheral blood were collected from three groups’ patients, the supernatant was centrifuged and the content of IFN-γ in serum was tested by direct chemoluminescence immunometric assay based on double antibody sandwich method (XMUMIC, China).

### Statistical Analysis

Statistical analysis was conducted using SPSS (version 24.0, SPSS,Chicago, IL). Median (quartile spacing) [M (Q)] for non-normal measurement data. The results of peripheral blood lymphocyte subsets (including activation indicator) and IFN-γ test between AT, LT and HCs groups were compared by Mann–Whitney U test. A P value of ≤ 0.05 was considered statistically significant.

## Results

### Changes in Serum IFN-γ Detection Levels in AT Group, LT Group and HCs Group [M (Q)] (pg/ml)

**Fig. 1:**
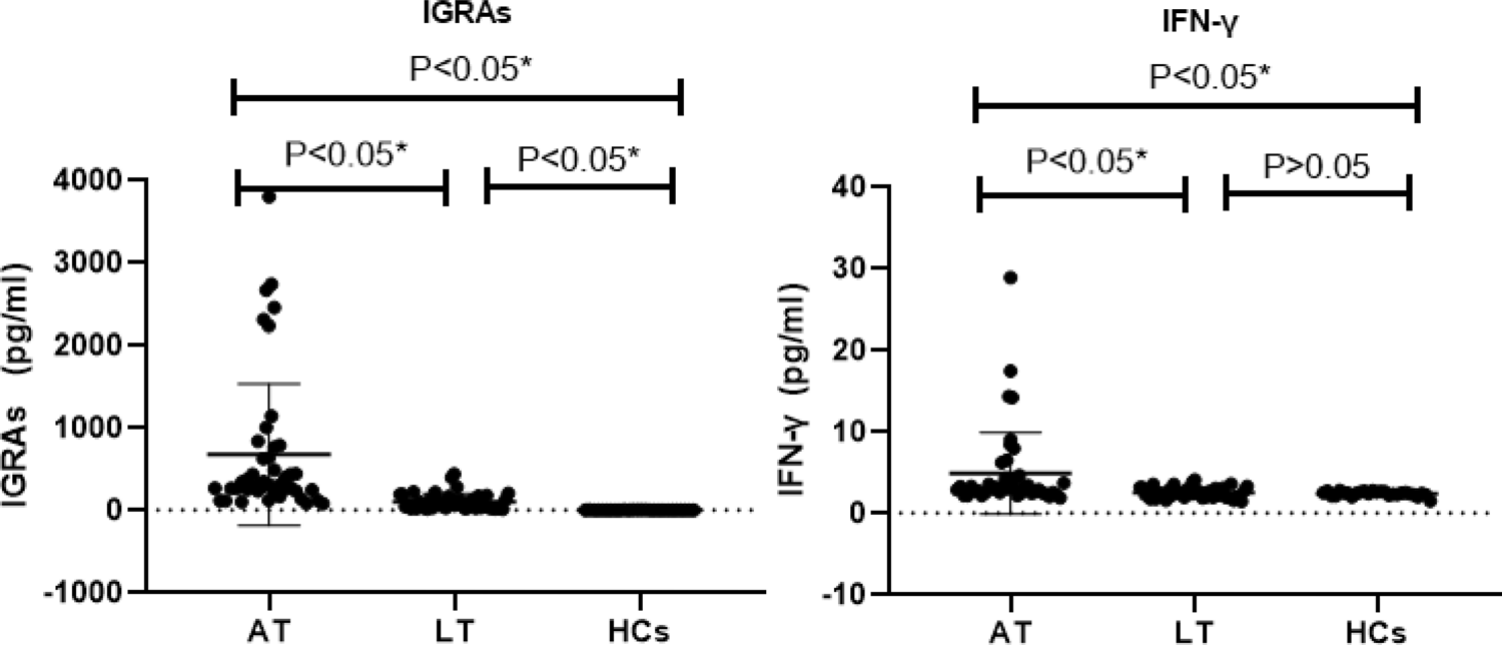
Comparison of the detection results of IGARs and IFN-γ in the three groups. *This indicates a statistical difference. P values were calculated by Mann–Whitney U test

This analysis was based on data with 45 AT group, 44 LT group and 32 HCs group. After a non-parametric test and analysis, The level of serum IFN-γ was significantly higher in the AT group [15.0 (1.77)] than in the LT group [2.45 (0.89)] and HCs [2.44 (0.36)] (*P* < 0.05), however, there were no statistical differences between the LT and HCs groups; The results of IGRAs in AT, LT and HCs groups were [316.24 (397.96)],[67.23 (121.19)] and [1.0 (3.75)], respectively, comparisons between the three groups were all* P* < 0.05; (Tables 2 and 3, Fig. 1).

**Table 2:** Results of lymphocyte subsets and IFN-γ between AT, LT and HCs groups [M (Q)]

	AT (*n* = 36)	LT (*n* = 31)	HCs (*n* = 32)
IGRAs pg/ml	316.24 (397.96) (*n* = 45)	67.23 (121.19) (*n* = 44)	1.0 (3.75)
IFN-γ pg/ml	3.15 (1.77) (*n* = 45)	2.45 (0.89) (*n* = 44)	2.44 (0.36)
NKT (CD3^+^CD16^+^CD56^+^) %	6.2 (5.0)	0.15 (0.45)	0.12 (0.1)
CD3^+^ T %	67.8 (11.9)	70.74 (16.52)	75.01 (9.7)
CD4^+^T %	35.15 (14.85)	38.58 (17.29)	44.08 (8.42)
CD8^+^ T %	24.15 (12.1)	23.12 (11.84)	25.63 (7.73)
CD4/CD8	1.64 (1.09)	1.39 (1.18)	1.60 (0.68)
Treg (CD4^+^CD25^+^CD127^−^) %	8.00 (2.55)	7.04 (2.11)	7.17 (1.79)
CD8^+^CD28^+^ T %	10.29 (6.98)	11.92 (7.34)	14.61 (8.44)
CD3^+^HLA-DR^+^ T %	2.95 (2.68)	2.52 (2.37)	1.79 (1.61)
CD4^+^HLA-DR^+^ T %	1.41 (1.5)	1.16 (1.54)	0.63 (0.19)
CD8^+^HLA-DR^+^ T %	1.19 (1.16)	0.95 (1.18)	0.95 (0.81)
NK (CD3^−^CD16^+^CD56^+^) %	9.86 (6.55)	13.65 (17.42)	10.33 (5.32)
CD16^+^CD56^+^CD69^+^ %	1.18 (2.29)	0.61 (1.25)	0.38 (0.44)

**Table 3:** Comparison of lymphocyte subsets and IFN-γ between AT, LT and HCs groups

	AT vs LT group		AT vs HCs group		LT vs HCs group	
	Z	P	Z	P	Z	P
IGRAs	− 6.548	0.000*	− 7.066	0.000*	− 7.036	0.000*
IFN-γ	− 4.018	0.000*	− 4.270	0.000*	− 2.900	0.772
NKT (CD3^+^CD16^+^CD56^+^)	− 6.587	0.000*	− 7.033	0.000*	− 0.503	0.615
CD3^+^ T	− 0.528	0.597	− 2.962	0.003*	− 1.504	0.12
CD4^+^ T	− 1.308	0.191	− 3.048	0.002*	− 1.431	0.153
CD8^+^ T	− 0.654	0.513	− 0.418	0.676	− 1.183	0.237
CD4/CD8	− 0.887	0.375	− 0.805	0.421	− 0.138	0.891
Treg CD4^+^CD25^+^CD127^−^)	− 1.390	0.165	− 2.673	0.008*	− 0.908	0.364
CD8^+^CD28^+^ T	− 1.610	0.107	− 3.159	0.002*	− 1.816	0.069
CD3^+^HLA-DR^+^ T	− 1.390	0.165	− 3.565	0.000*	− 2.063	0.039*
CD4^+^HLA-DR^+^ T	− 1.188	0.235	− 5.188	0.000*	− 3.524	0.000*
CD8^+^HLA-DR^+^ T	− 1.038	0.299	− 1.432	0.152	− 0.124	0.901
NK (CD3^−^CD16^+^CD56^+^)	− 1.346	0.178	− 1.561	0.119	− 0.206	0.837
CD16^+^CD56^+^CD69^+^	− 1.044	0.396	− 2.349	0.019*	− 0.812	0.417

^*^This indicates a statistical difference

### Changes in Lymphocyte Subsets Levels in AT Group, LT Group and HCs Group [M (Q)] (%)

**Fig. 2:**
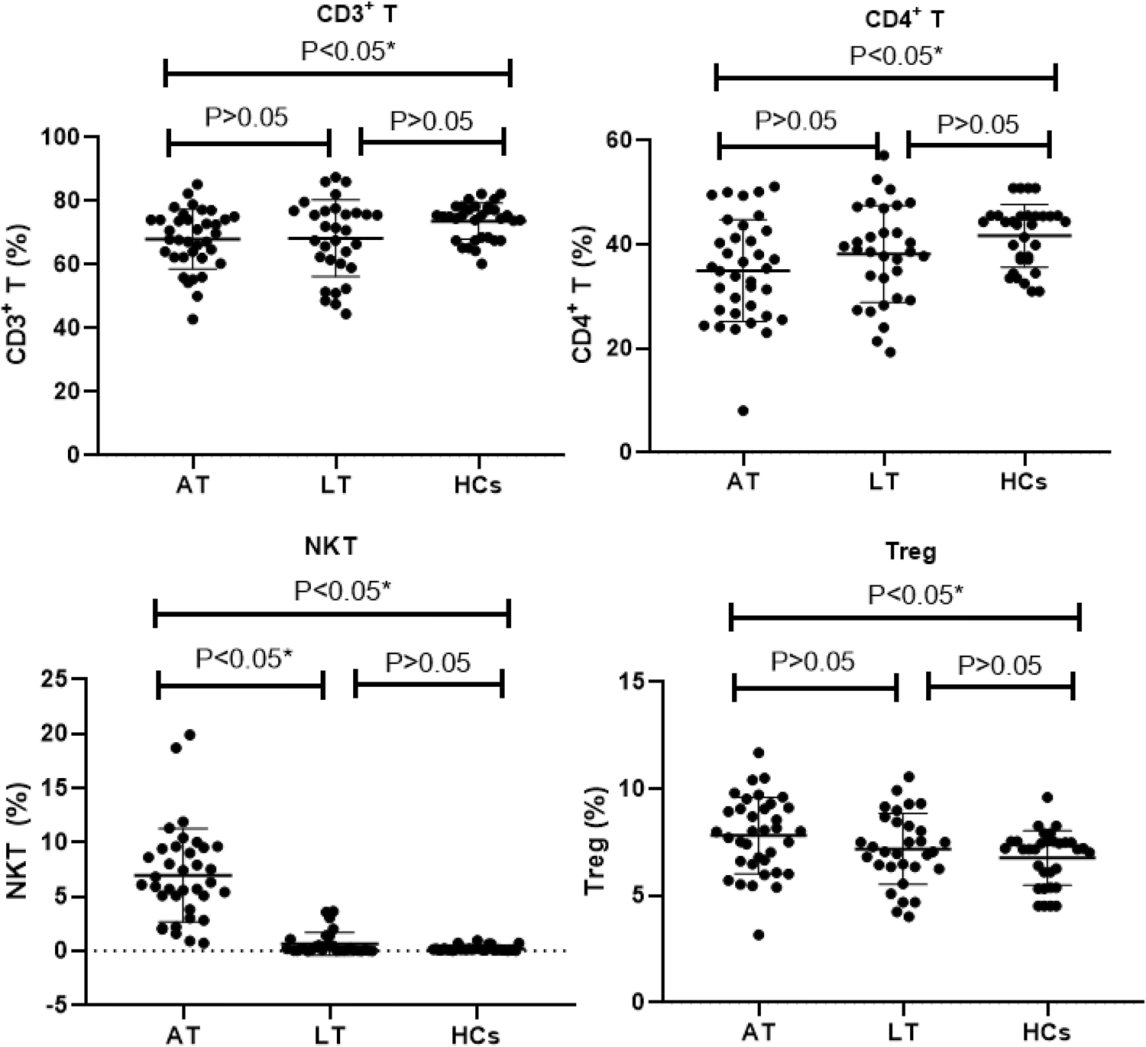
Comparison of the results of lymphocyte subsets in the three groups. *This indicates a statistical difference. P values were calculated by Mann–Whitney U test

This analysis was based on data with 36 AT group, 31 LT group and 32 HCs group. After a non-parametric test and analysis, the results of lymphocyte subsets analysis showed that the percentage of NKT cells was significantly higher in AT group [6.2 (5.0)] than in LT [0.15 (0.45)] and HCs groups [0.12 (0.1)] (*P* < 0.05); The percentages of CD3^+^ T cells [67.8 (11.9)] and CD4^+^ T cells [35.15 (14.85)] were significantly lower in AT group than in HCs group (*P* < 0.05), and the percentage of Treg cells [(8.00 (2.55)] was significantly higher in AT group than in HCs group (*P* < 0.05), those with no statistical difference between AT and LT group as well as between LT and HCs group.( Tables 2 and 3, Fig. 2).

### Changes in Activated Lymphocytes Indicator Levels in AT Group, LT Group and HCs Group [M(Q)] (%)

**Fig. 3:**
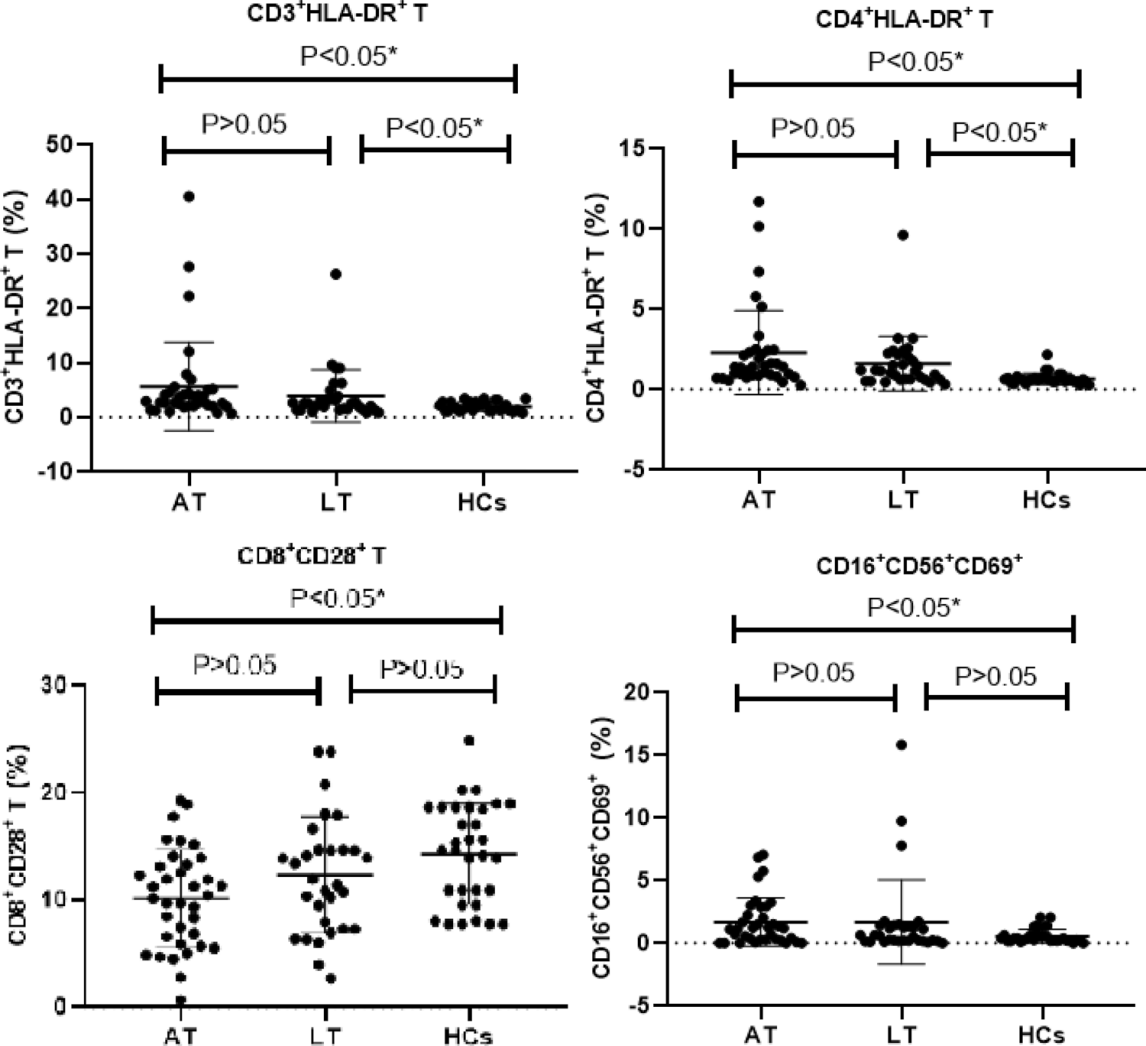
Comparison of the results of the lymphocyte activation indicators in the three groups. *This indicates a statistical difference. P values were calculated by Mann–Whitney U test

This analysis was based on data with 36 AT group, 31 LT group and 32 HCs group. After non-parametric test analysis, the results of activated lymphocytes showed that the percentage of CD16^+^CD56^+^CD69^+^ cells was significantly higher in AT group [1.18 (2.29)] than in HCs group [0.38 (0.44)] (*P* < 0.05), with no statistical difference from the LT group; The percentages of CD3^+^HLA-DR^+^T cells and CD4^+^HLA-DR^+^T cells were significantly higher in AT group [2.95 (2.68), 1.41 (1.5)] as well as LT group [2.52 (2.37),1.16 (1.54)] than in HCs group [1.79 (1.61),0.95 (0.81)] (*P* < 0.05), while AT group with no statistical difference from the LT group. The percentage of CD8^+^CD28^+^ T cells was significantly lower in AT group [10.29 (6.98)] than in HCs group (*P* < 0.05) (Tables 2 and 3, Fig. 3).

### ROC Curve Analysis of Measurements in the Diagnosis of Active Pulmonary Tuberculosis

**Fig. 4:**
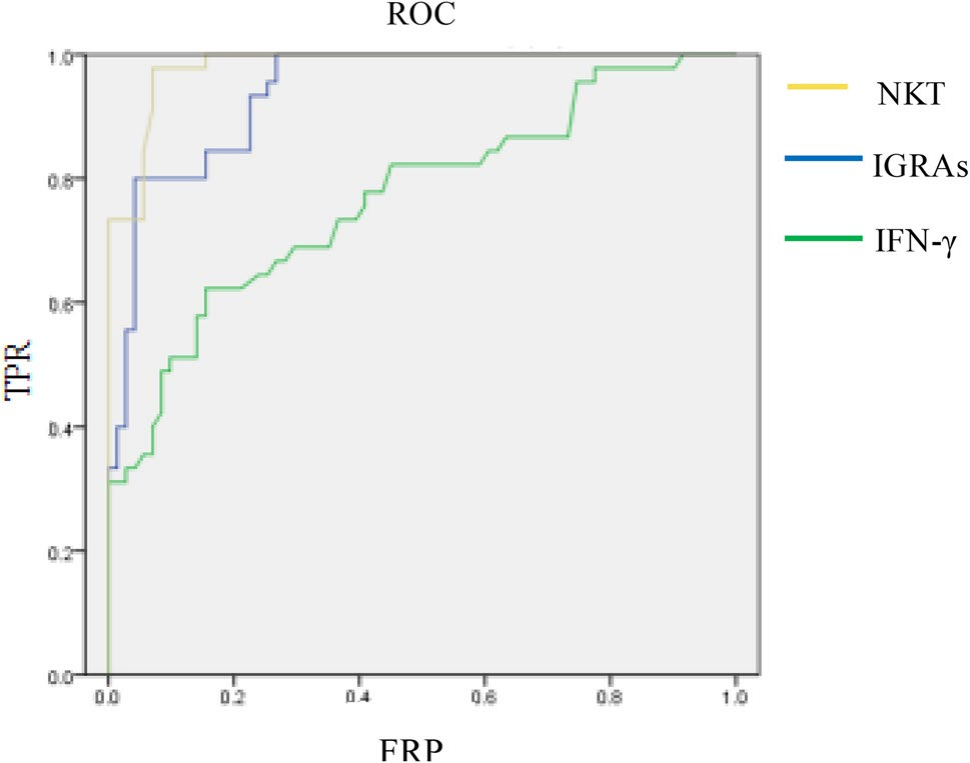
The ROC curve of NKT, IGRAs and IFN-γ in the diagnosis of active pulmonary tuberculosis were 0.970, 0.940 and 0.768, respectively, all P < 0.001

This analysis was based on data with 45 AT group, 44 LT group and 32 HCs group. The areas under ROC curve of NKT cells, IGRAs and serum IFN-γ in the diagnosis of active pulmonary tuberculosis were 0.970, 0.940 and 0.768, respectively, all *P* < 0.001. (Fig. 4).

## Discussion

*MTB* is an intracellular parasite. After *MTB* infection, it first induces innate immunity which is phagocytic by macrophages. After apoptosis, the phagocytic cells release a large number of cytokines (such as IFN-γ) and *MTB* specific antigens, which are presented to lymphocytes by antigen-presenting cells and induced acquired immunity [15]. Both lymphocytes and cytokines play important roles in *MTB* infection.

IGRAs, widely recommended by WHO and CDC [12], failed to distinguish between the active and latent stages of TB, but studies have shown that IGRAs plays an important role in predicting the probability of TB infection developing active TB [16]. In this study, direct detection of serum IFN-γ and IGRAs in the three groups found that the level of IFN-γ was significantly higher in the AT group than in the LT and HCs groups (*P* < 0.05), however, there were no statistical differences between the LT and HCs groups; The results of IGRAs showed significantly higher in AT group than LT group as well as higher in LT than HCs group. Also ROC curve analysis, the AUCs of IGRAs and IFN-γ in the diagnosis of active pulmonary tuberculosis were 0.940 and 0.768, respectively, all *P* < 0.001. Therefore, IGRAs not only has good diagnostic efficiency for ATBI, but also can be combined with direct detection of serum IFN-γ as a laboratory method to distinguish ATBI from LTBI and healthy people.

The NKT cells have both the phenotype and function of the NK cells and the T cells. Since the NK cells mainly participate in the innate immunity, and the T lymphocytes mainly participate in the adaptive immune response, so the NKT cells have became a bridge between the innate immunity and the adaptive immunity [17]. After NKT cells infected *MTB* in the body, activated NKT cells inhibit tuberculosis proliferation and effectively kill *MTB* by releasing a series of cytokines such as IFN-γ [18]. Our study showed that the percentage of NKT cells was significantly higher in both AT groups than in LT and HCs groups, with no statistical difference between LT and HCs groups. And the ROC curve of NKT in the diagnosis of active pulmonary tuberculosis was AUC = 0.970. *P* < 0.001. The results showed that NKT cells had a grate diagnostic efficiency in the ATBI.

Other results of lymphocyte subsets showed that the percentages of CD3^+^ T, CD4^+^ T and CD8^+^ T cells were significantly lower in AT group than in HCs group, and the percentage of Treg cells was significantly higher in AT group than in HCs group, and these all with no statistical difference between the LT group and HCs group. The CD4^+^ T cells play a major role in the anti-tuberculosis immune response [19]. Depending on the function, the CD4^+^ T cells were divided into different cell subpopulations, including Th1, Th2, Treg, and Th17 cells. After activating, the CD4^+^ T cells proliferate and produce cytokines, which can reactivate macrophages and inhibit the growth of *MTB* [20]. The results of this study showed that the ATBI have impaired immune function, and mainly have reduced CD4^+^ T cells, which consistent with the anti-tuberculosis immunological response effect.

After human infected with *MTB*, Treg cells can have a protective inhibition effect on the immune response of T lymphocytes, and reduce the immune damage caused by strong immune response [21]. However, when the body’s immune function is disordered, the surface molecules of Treg cells such as cytotoxic T lymphocyte-associated antigens will inhibit CD4^+^T and CD8^+^T cells activation, thus attenuated the macrophage clearance on *MTB* leading to the development of tuberculosis [22]. The results of the increased percentages of Treg cells in AT group were consistent with the tuberculosis immune response.

The results of lymphocyte activation indicators showed that the percentages of CD3^+^HLA-DR^+^T and CD4^+^HLA-DR^+^T were significantly higher in AT group as well as LT group than in HCs group, while AT group with no statistical difference from the LT group. The percentage of CD16^+^CD56^+^CD69^+^ was significantly higher in AT group than in HCs group, with no statistical difference from the LT group. HLA-DR are usually expressed on the surface of activated T lymphocytes, and increased expression of HLA-DR predicted activation of the immune system [23], CD69 is also one of the indicators of the early activation of T lymphocytes [24]. The results of this study showed that although the CD3^+^ T and CD4^+^T did not change significantly in the LT group, the activation indicators of CD3^+^HLA-DR^+^ and CD4^+^HLA-DR^+^ can effectively distinguish the LT group from the HCs group. NK (CD16^+^CD56^+^) cells have been implicated in the early immunity of intracellular pathogens [25]. In this experiment, the NK cell did not change significantly in the AT group, but the activated NK cells (CD16^+^CD56^+^CD69^+^) were significantly increased in AT group. The results still indicate that NK cells play an important role in controlling tuberculosis infection in ATBI.

CD8^+^CD28^+^T cells can directly kill the bacteria and induce apoptosis in their infected target cells, and research has shown that CD8^+^CD28^+^T cell decreased during periods of inflammatory activity [26], this was consistent with our findings. CD28^+^ acts as a second signal of T cell activation to promote the further proliferation of the cells activated by the first signal and to secrete the corresponding cytokines for immune effects [27]. Therefore, the second signal of CD28 decreased expression which causing weakened T lymphocyte activation in patients with active tuberculosis, it may be one of the mechanisms of immune escape in this bacterium.

## Conclusion

In conclusion, combined IGRAs, serum IFN-γ and NKT cells not only has good diagnostic efficiency for the ATBI, but also provides a laboratory diagnostic method to distinguish ATBI from LTBI. Activation indicator of CD3^+^HLA-DR^+^T and CD4^+^HLA-DR^+^T cells can effectively distinguish LTBI from healthy people. while combined CD3^+^T, CD4^+^T, CD8^+^CD28^+^T, Treg and CD16^+^CD56^+^CD69^+^ cells can distinguish ATBI from healthy people. Therefore, the combined direct detection of serum IFN-γ and IGRAs as well as lymphocyte subsets with activation indicators can provide laboratory basis for the diagnosis and differential diagnosis of active tuberculosis and *MTB* latent infection.
